# Perceptions and Attitudes of Emergency Physicians in Saudi Arabia Regarding Medical Use of Artificial Intelligence

**DOI:** 10.7759/cureus.98583

**Published:** 2025-12-06

**Authors:** Khalid Munawir Alotaibi, Mohammed Ibraheem Aljuaid, Salman Hamad Alharbi, Amal Alyahya

**Affiliations:** 1 Department of Emergency Medicine, Ministry of National Guard Health Affairs, Riyadh, SAU; 2 Department of Medical Specialties Medicine, College of Medicine, Majmaah University, Majmaah, SAU; 3 College of Medicine, King Saud Bin Abdulaziz University for Health Sciences College of Medicine, Riyadh, SAU

**Keywords:** artificial intelligence, digital health, emergency medicine, physician attitudes, saudi arabia

## Abstract

Background: Artificial intelligence (AI) is increasingly recognized for its potential to support clinical decision-making and workflow optimization in emergency medicine (EM). However, the perspectives of emergency physicians, particularly in Saudi Arabia, regarding its integration remain understudied.

Objective: To assess the attitudes, familiarity, perceived advantages, and concerns of Saudi emergency physicians regarding the application of AI in clinical emergency practice.

Methods: A descriptive cross-sectional study was conducted between February and April 2025 using a self-administered online questionnaire distributed to emergency physicians across Saudi Arabia. Inclusion criteria were active clinical engagement and at least one year of EM experience. Descriptive and inferential statistics were performed using SPSS version 26.

Results: Among 101 participants, the majority were male, 71 (70.3%), and aged 25-29 years, 71 (70.3%). A total of 51 (50.5%) agreed that they were familiar with AI, while 97 (96%) believed that AI has useful applications in medicine. However, 41 (40.6%) disagreed that AI has superior diagnostic ability compared to human physicians, while 35 (34.7%) strongly disagreed that AI could replace their roles. The most cited advantages of AI were access to high-quality real-time clinical data, 75 (74.3%), absence of emotional or physical exhaustion, 69 (68.3%), and faster healthcare processes, 64 (63.4%). In cases of disagreement between physician and AI, 95 (94.1%) preferred the physician's opinion. Key concerns included AI's unreliability in unpredictable situations, 72 (71.3%), lack of flexibility in application, 64 (63.4%), and inability to handle controversial topics, 69 (68.3%). Over half, 54 (53.47%), expressed positive attitudes toward AI in EM. Attitudes were significantly more positive among physicians from the northern region (β=2.8, 95% CI: 0.37-5.1, p=0.024) and western region (β=2.0, 95% CI: 0.30-3.7, p=0.022) compared to the central region.

Conclusion: Saudi emergency physicians generally express positive attitudes toward AI, particularly in supporting clinical tasks, but remain cautious about its diagnostic authority and legal implications. Regional variation suggests the need for tailored educational and policy strategies to promote AI adoption in emergency settings.

## Introduction

Artificial intelligence (AI) is the field of study and application of developing computer systems that can handle issues that typically need human decision-making. In the field of Emergency Medicine (EM), professionals acknowledge that AI will have a significant influence on patient care [1,2]. EM is a unique field that deals with patients of all ages who have both acute and subacute conditions, and who have not yet been diagnosed. Due to the diverse nature of the Emergency Department (ED), there are several possible methods by which AI might enhance treatment. In disciplines that include a broad range of knowledge, such as EM, it is not always clear what the most valuable applications of AI will be in the near future [2]. A growing number of research articles and scoping studies that detail AI solutions created especially for use in the ED have been released in recent years. These include boosting the efficiency and accuracy of triage, diagnosis, and prognosis of various diseases or clinical syndromes, supporting targeted medication administration, and improving the interpretation of medical imaging. They also include improving patient safety through AI-powered patient monitoring [3,4].

Although clinical AI tools have seen significant progress, there are several barriers that hinder the use of AI technology in the field of medicine. These concerns include questions about accountability for medical errors caused by AI, public opinion, legal oversight, and the lack of transparency in understanding how AI arrives at its findings, often known as the "black-box" phenomenon or lack of "explainability" [1]. Furthermore, when it comes to the adoption of these applications, there is a noticeable absence of involvement from medical experts in both the initial evaluation of requirements and the subsequent design process [5,6]. To overcome this constraint, qualitative surveys have been carried out in medical fields other than EM to evaluate the requirements of healthcare personnel who might gain from the use of new AI technologies [7,8]. These investigations specifically explore the comprehension and concerns of doctors regarding the technology, measure their anticipations, and pinpoint requirements that might steer the advancement of AI tools.

There is currently no existing needs analysis that is analogous to the one conducted on the application of AI in EM. Multiple literature studies provide an overview of the present advancements and uses of AI in EM fields while also discussing its possible future advantages [9,10]. However, there is a lack of information on the present use of AI by emergency physicians, their comprehension of this technology, and most significantly, their preferences for the integration of AI into the clinical workflow and the areas they feel AI development should be prioritized.

The future influence of AI on emergency care is anticipated, with potential uses such as predicting patient outcomes, early detection of deterioration via vital sign monitoring, and analyzing clinical images [11]. Nevertheless, the integration of novel technologies in the healthcare environment is complex, since the reception of these technologies varies across individuals and organizations [12].

The aim of this study was to evaluate the attitudes of Saudi emergency physicians toward AI and its implementation in the field of emergency medicine. The primary objective is to assess their familiarity with AI, as well as their perceived advantages, concerns, and overall views regarding the integration of AI into clinical emergency practice.

## Materials and methods

Study design and population

This is a descriptive cross-sectional study, performed using a self-administered online questionnaire from February to April 2025. The targeted population was emergency physicians from all over the Kingdom of Saudi Arabia with at least one year of experience in the field, who were actively practicing and involved in clinical duties. Exclusion criteria included Saudi physicians who were practicing abroad, nonclinically active physicians, and incomplete responses. A convenience sampling approach was used, and the survey link was distributed through national emergency medicine networks and professional online physician groups across all regions of Saudi Arabia. The minimum required sample size was 350 participants based on the calculation used by OpenEpi. The population size of emergency physicians in Saudi Arabia was considered approximately 3850 physicians (according to the Statistical Yearbook by the Ministry of Health, 2018). In addition, the confidence interval level was considered 95%, with an anticipated frequency percentage of 50%.

Tool structure and validity

A self-administered online questionnaire was used for this study, which was inspired by and retrieved from previous research projects published in the literature [7,8]. The questionnaire consists of the following sections: (i) demographic characteristics of the participants, including age, gender, and region of practice; (ii) general assessment of AI familiarity, which included five Likert scale questions with the following five options: Strongly Agree, Agree, Neutral, Disagree, and Strongly Disagree; and (iii) perceptions of AI use in clinical practice, which included several questions in various formats such as open questions, multiple-choice questions, and multiple-response questions. The questionnaire underwent face validation by three emergency medicine experts and was piloted with 50 emergency physicians to ensure clarity and content validity before distribution. Then, it was distributed to the participants in the original language (English).

Statistical analysis

Data were collected, reviewed, and cleaned in Microsoft Excel (Microsoft Corporation, Redmond, Washington), then analyzed using IBM SPSS Statistics for Windows, Version 26 (Released 2019; IBM Corp., Armonk, New York). All statistical methods used were two-tailed with an alpha level of 0.05, and significance was considered if the p-value was less than or equal to 0.05. Descriptive analysis was used for the study. Categorical data were reported as frequencies and percentages, while numerical data, such as age, were reported as mean ±SD. The total attitude score was calculated, and those who scored the mean value or above were considered to have positive attitudes. Wilcoxon rank sum and Kruskal-Wallis rank sum tests were used to assess the association between demographic characteristics and attitude toward the use of AI. A p-value less than 0.05 was considered statistically significant.

## Results

Participant demographics

As indicated in Table 1, the study involved 101 emergency physicians. Seventy-one participants were male, 71 (70.3%), 71 (70.3%) were 25-29 years old, and more than half, 58 (57.4%), practiced medicine in the central region of Saudi Arabia. 

**Table 1:** Demographic Characteristics of the Included Participants (N = 101) ^§^Average age in years ± SD. ^#^Age presented in groups by years.

Factors	Category	Values
Age^§^		28±2.69
Age^#^, Freq (%)	25-29 years	71 (70.3%)
	30-34 years	26 (25.7%)
	≥35 years	4 (4.0%)
Gender, Freq (%)	Male	71 (70.3%)
	Female	30 (29.7%)
Region of practice, Freq (%)	Central region	58 (57.4%)
	Western region	18 (17.8%)
	Southern region	12 (11.9%)
	Northern region	8 (7.9%)
	Eastern region	5 (5.0%)

Familiarity and general attitudes toward AI

**Figure 1:**
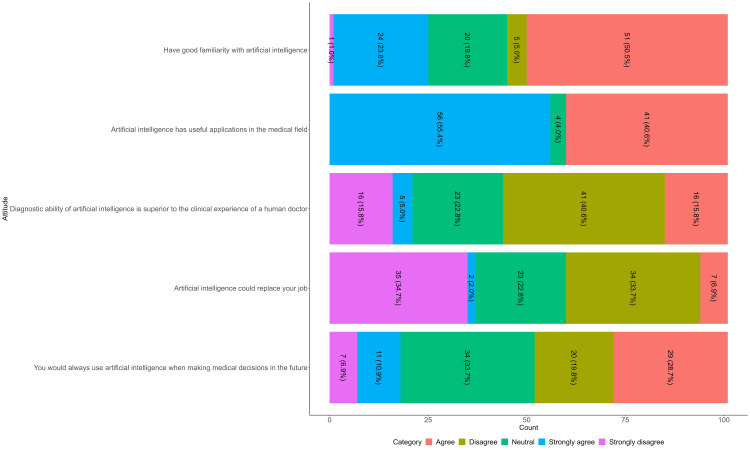
Familiarity and General Attitudes Toward AI Among Emergency Physician in Saudi Arabia, 2025

In terms of general attitudes toward AI, just under half of the participants agreed that they were familiar with AI, 51 (50.5%), with physicians reporting strong agreement at the next level, 24 (23.8%). The majority of participants agreed or strongly agreed about AI's useful applications in the medical field, 41 (40.6%) and 56 (55.4%), respectively. Nearly half, 41 (40.6%), disagreed that AI had greater diagnostic ability than the clinical experience of a human doctor, while 35 (34.7%) strongly disagreed. In addition, 34 (33.7%) disagreed that AI would replace their job. The majority of the sample, 34 (33.7%), reported being neutral about their consistent use of AI in making medical decisions in the future (Figure 1).

Perceptions of AI use in clinical practice

The participants’ perceptions of AI use in clinical practice are shown in Table 2. More than three-quarters, 75 (74.3%), of participants agreed that the most recognized advantage of AI was that it could provide high-quality, clinically relevant data very quickly in a real-time situation. This was followed by 69 (68.3%) who recognized that AI faced no emotional fatigue or physical limits, and 64 (63.4%) who recognized that AI could be useful in expediting processes in healthcare. Among the other advantages mentioned were reporting AE and SAE to help reduce medical errors, 58 (57.4%), and no space-time limitations, 50 (49.5%). When asked whose opinion they would prefer in the event of a disagreement between the physician and AI, 95 (94.1%) chose the physician, while only 2 (2.0%) selected the opinion of the AI. Only 4 (4.0%) selected the opinion of the patient. When asked in which provinces AI is most useful, there were several responses. A total of 72 (71.3%) selected biopharmaceutical and their R&D, 61 (60.4%) selected diagnosis, 54 (53.5%) selected providing medical aid to underprivileged areas, 45 (44.6%) selected developing social insurance programs, 23 (22.8%) selected treatment decisions, and 10 (9.9%) selected direct treatment including surgery. Participants also expressed caution regarding the subject of AI in medicine. The greatest concern was that AI is unable to provide reliable opinions in uncertain situations due to insufficient information, with 72 (71.3%) selecting this option. This was followed by difficulty in addressing controversial subjects, 69 (68.3%), lack of flexibility in applying AI to every patient, 64 (63.4%), development by specialists with little clinical experience, 49 (48.5%), and low ability to sympathize with and consider the emotional well-being of patients, 42 (41.6%).

**Table 2:** Perceptions of AI Use in Clinical Practice Among Emergency Physician in Saudi Arabia, 2025 ^§^As this was a multiple-choice question, percentages represent the proportion of participants who selected each option, rather than totaling 100%.

Question	No	%^§^
What are the Advantages of using artificial intelligence?		
AI can speed up processes in health care	64	63.4%
AI can help reduce medical errors	58	57.4%
AI can deliver vast amounts of clinically relevant high-quality data in real time	75	74.3%
AI has no space-time constraint	50	49.5%
AI has no emotional exhaustion nor physical limitation	69	68.3%
If your medical judgment and an artificial intelligence’s judgments differ, which will you follow?		
Doctor’s opinion	95	94.1%
Artificial intelligence’s opinion	2	2.0%
Patients’ choice	4	4.0%
In which field of medicine do you think artificial intelligence will be most useful?		
Making a diagnosis	61	60.4%
Making treatment decisions	23	22.8%
Direct treatment (including surgery)	10	9.9%
Biopharmaceutical research and development	72	71.3%
Providing medical assistance in underserved areas	54	53.5%
Development of social insurance program	45	44.6%
What are you concerned about the application of AI in medicine?		
It cannot be used to provide opinions in unpredicted situations due to inadequate information	72	71.3%
It is not flexible enough to be applied to every patient	64	63.4%
It is difficult to apply to controversial subjects	69	68.3%
The low ability to sympathize and consider the emotional well-being of the patient	42	41.6%
It is developed by a specialist with little clinical experience in medical practice	49	48.5%

**Figure 2:**
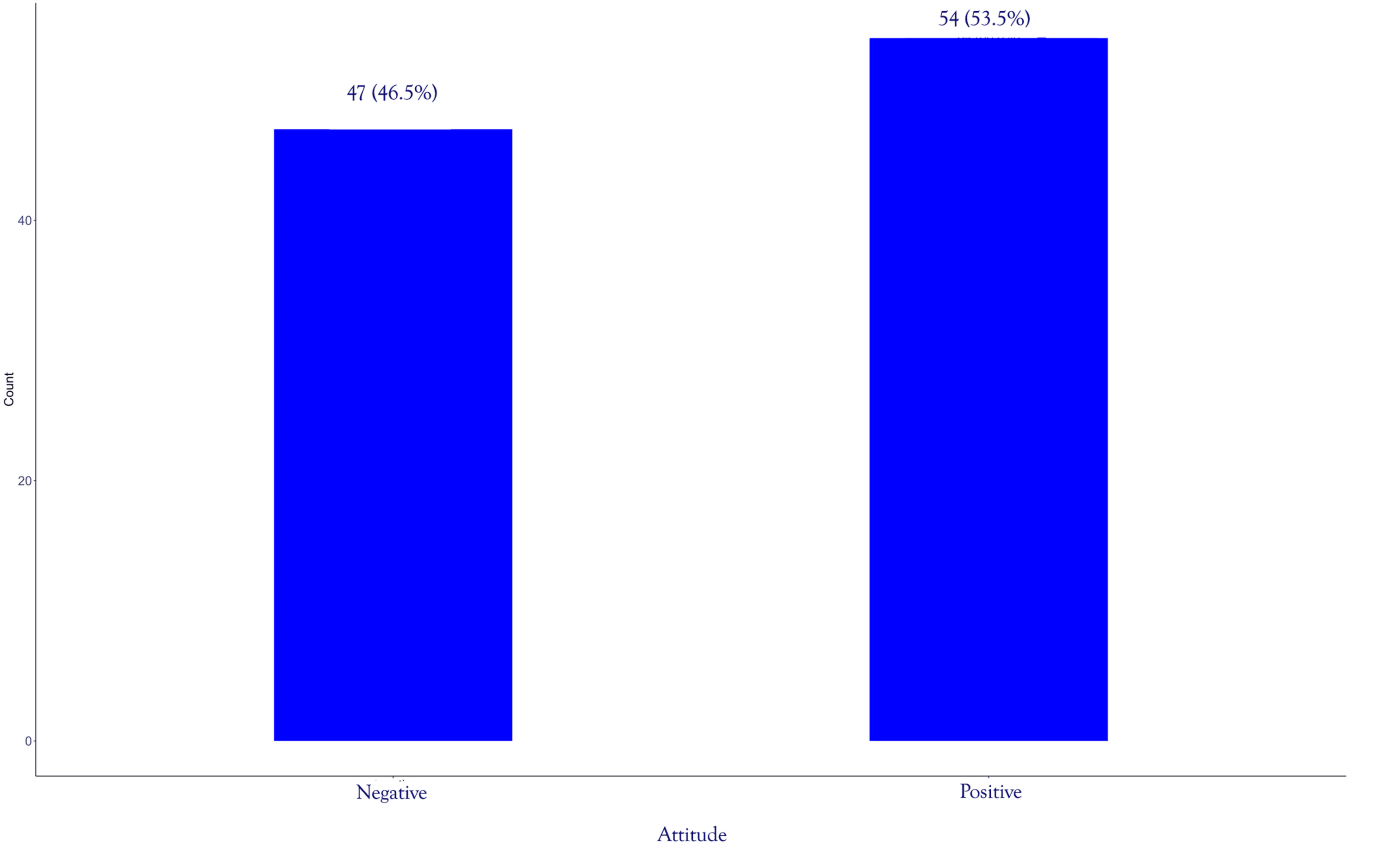
Attitude Level Toward Artificial Intelligence Use in Emergency Medicine

More than half, 54 (53.47%), had a positive attitude toward the use of AI in EM, as shown in Figure 2.

**Figure 3:**
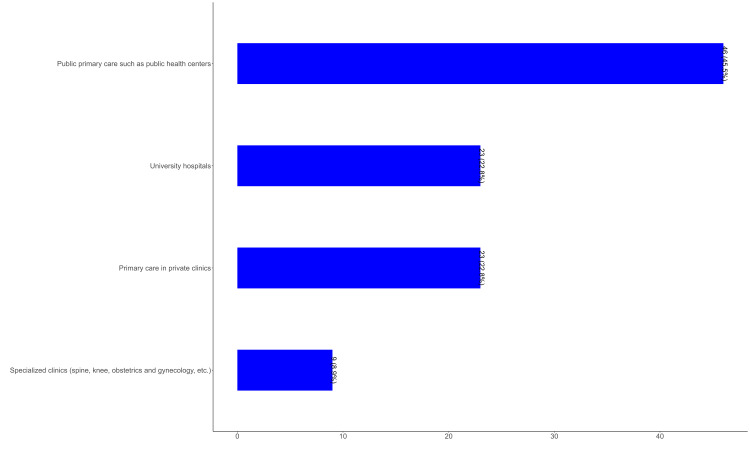
Distribution of Emergency Physicians' Opinions on the First Healthcare Sector to Commercialize Artificial Intelligence, 2025

Figure 3 illustrates the distribution of EM physicians' opinions regarding the healthcare sector most likely to commercialize AI first. A significant proportion of participants, 46 (45.5%), identified public primary care settings, such as public health centers, as the most probable entry point for AI commercialization. This was followed by private primary care clinics and university hospitals, each selected by 23 (22.8%) of respondents. In contrast, only 9 (8.9%) of participants believed that specialized clinics, such as spine, obstetrics, and gynecology clinics, would lead the commercialization of AI in healthcare.

**Figure 4:**
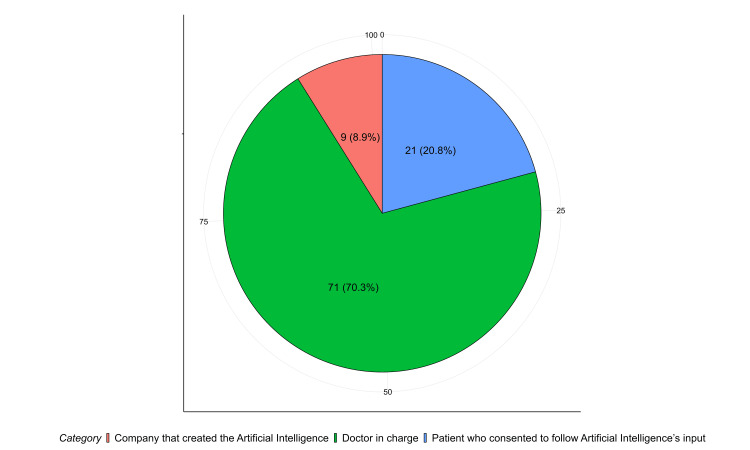
Perceptions of Legal Liability for Issues Arising From the Use of Artificial Intelligence in Medicine According to Emergency Physicians' Opinions, 2025

Figure 4 presents participants' views on who should bear legal responsibility for issues arising from the use of AI in medical practice. A substantial majority, 71 (70.3%), believed that the doctor in charge should be held legally accountable. In contrast, 21 (20.8%) attributed liability to the company that developed the AI, and a smaller proportion, 9 (8.9%), held the patient who consented to follow AI-generated recommendations responsible.

Only regions of practice were significantly associated with attitude score, with those from northern regions reporting higher attitude scores than other regions (p=0.024) (Table 3).

**Table 3:** Association Between Demographic Characteristics and Attitude Score ^1^Attitude score: mean±SD. ^2^Wilcoxon rank sum test; Kruskal-Wallis rank sum test. ^3^Holm correction for multiple testing.

Characteristics	N = 101^1^	p-value^2^	q-value^3^
Gender		0.5	0.5
Female	15.8±2.7	-	-
Male	16.4±3.2	-	-
Age group in years		0.2	0.5
25-29	15.9±2.9	-	-
30-34	16.6±3.5	-	-
35-37	18.3±3.0	-	-
Region of practice		0.008	0.024
Central	15.6±2.7	-	-
Eastern	13.6±3.3	-	-
Northern	18.0±1.4	-	-
Southern	17.2±3.1	-	-
Western	17.4±3.8	-	-

Participants from northern regions (Beta: 2.8, 95% CI: 0.37, 5.1, p=0.024) and western regions (Beta: 2, 95% CI: 0.30, 3.7, p=0.022) had significantly higher positive attitudes than those from the central region (Table 4).

**Table 4:** Determinants of Attitudes Toward AI Use Among Emergency Physicians ^1^CI: confidence interval.

Characteristics	Beta	95% CI^1^	p-value
Gender			
Female	—	—	
Male	0.86	-0.50, 2.2	0.2
Age group in years			
25-29	—	—	
30-34	-0.37	-1.9, 1.2	0.6
35-37	1.7	-1.6, 5.0	0.3
Region of practice			
Central	—	—	
Eastern	-1.5	-4.4, 1.3	0.3
Northern	2.8	0.37, 5.1	0.024
Southern	1.6	-0.59, 3.8	0.2
Western	2.0	0.30, 3.7	0.022

## Discussion

AI applications in EM have significantly expanded over the past decade. AI offers a unique value proposition for emergency physicians by enhancing clinical decision-making in real time [13]. This study aimed to assess the perceptions and attitudes of emergency physicians in Saudi Arabia toward the medical use of AI.

Our findings show that over half, 54 (53.47%), of the emergency physicians demonstrated a positive attitude toward AI, with the most commonly recognized advantage being its ability to process vast amounts of clinically relevant, high-quality data. Conversely, the main concern reported was AI's inability to provide reliable decisions in unpredictable situations due to insufficient information, as reported by 72 (71.3%).

In our study, 54 (53.47%) of emergency physicians expressed a positive attitude. In contrast, a previous Saudi study reported that only 8.8% of physicians supported AI, with the majority uncertain about its use [14]. This shift in perception is promising and indicates progress toward greater acceptance. Additionally, 75 (74.3%) of participants in our study reported familiarity with AI, whereas the earlier study noted that 74.1% had never used such systems [14]. Another study from Saudi Arabia found that slightly more than half of healthcare professionals were familiar or very familiar with AI [15]. The high familiarity rate in our study supports the potential for smoother integration of AI into emergency medical practice.

Almost all respondents, 97 (96%), acknowledged the usefulness of AI in the medical field. Despite this, only 5.88% and 8.82% of major hospitals in Saudi Arabia have established AI-specialized centers or implemented AI in clinical care, respectively [16]. This highlights the gradual but promising transformation of healthcare through AI in the Kingdom.

However, only 40 (39.6%) of respondents agreed or strongly agreed that they would always use AI in future medical decisions. This rate is lower than anticipated and suggests the need for further initiatives to encourage AI adoption. While only 9 (8.9%) of participants believed that AI could replace their roles, the majority disagreed. Despite ongoing debate, AI is not intended to replace physicians but rather to enhance their roles and improve efficiency [17]. A 2017 Pew Research Center survey revealed that 72% of the public expressed concern, more than double the 33% who felt enthusiastic, about a future where robots and computers perform many human jobs [18]. Nevertheless, physicians will remain essential for handling ambiguous cases, performing physical examinations, and integrating complex clinical histories.

A significant association was found between younger age and the belief that AI could replace physicians, with those aged 25-29 years being more likely to hold this view. This may reflect greater exposure to advanced technology and a stronger awareness of AI’s capabilities in healthcare [19].

Regarding perceptions of AI in clinical practice, 75 (74.3%) of participants cited real-time access to large volumes of high-quality clinical data as the top advantage, followed by the absence of emotional exhaustion and physical limitations, 69 (68.3%). Another frequently reported benefit was improved efficiency in healthcare processes. These advantages have been documented in the literature [20-22]. On the other hand, the main concern was AI’s unreliability in unpredictable scenarios due to insufficient information, followed by its perceived lack of flexibility in individualized patient care. These concerns mirror those found in a study among Korean physicians [22].

Most participants, 72 (71.3%), identified biopharmaceutical research and development as the primary domain benefiting from AI. In Saudi Arabia, AI-related publications in healthcare have been rising steadily, with 8 indexed in PubMed, 10 in Cureus, and 14 in Google Scholar in 2023 alone [16]. Additionally, 61 (60.4%) of our respondents reported that AI is beneficial in supporting diagnosis. A systematic review found that machine learning models outperform traditional emergency severity scores in diagnosing and managing patients early [23]. These two domains, diagnosis and research, were also top-ranked by physicians in a study from Kuwait [24].

Nearly half of our participants, 46 (45%), believed that public primary care facilities, such as public health centers, would be the first to commercialize AI, ahead of university hospitals, 23 (22.8%), and private clinics, 23 (22.8%). A multicenter study from Kuwait found university hospitals (33.1%) and governmental PHC centers (32.5%) to be leading candidates for early AI implementation, followed by specialized clinics [24]. This reflects a shared regional perspective that publicly funded, high-volume healthcare settings are best positioned to lead AI integration due to government support, access to large datasets, and broad population coverage.

Regarding legal accountability in AI-related issues, 70% of emergency physicians believed that the responsible physician should remain legally accountable. This finding aligns with a Korean study [10]. Currently, there is no clear delineation of responsibility between healthcare providers, AI developers, and regulators in cases where AI errors lead to patient harm. Therefore, comprehensive policies are urgently needed to define responsibility and safeguard patient welfare [25].

Emergency physicians in the Northern and Western regions of Saudi Arabia had significantly higher attitude scores compared to those in the Central region. Further investigation is warranted to explore the underlying factors contributing to these regional differences.

This study is the first to explore the attitudes and perceptions of AI in medical practice, specifically among emergency physicians. Focusing on this group adds strength to the research, as emergency physicians work in fast-paced, high-stakes environments that require rapid clinical decision-making and adaptability, conditions under which AI can offer meaningful support. Furthermore, the inclusion of participants from various regions across Saudi Arabia enhances the generalizability of the findings. However, the study is not without limitations, including a smaller-than-required sample size and the use of convenience sampling, which may introduce selection bias. Moreover, the use of a self-administered questionnaire introduces the possibility of recall bias and social desirability bias. Additionally, the online nature of the survey may have limited participation to individuals with internet access during the data collection period. The cross-sectional design also restricts the ability to establish causal relationships. These factors limit the generalizability of the findings. Although the calculated minimum sample size was 350, only 101 responses were obtained. This reduces statistical power but still provides valuable insight into national trends among emergency physicians in Saudi Arabia.

## Conclusions

AI plays a central role in Saudi Arabia’s Vision 2030. A report by McKinsey & Co. suggests that digital health and AI technologies could contribute as much as USD 27 billion to the country’s healthcare economy by 2030. This study revealed that nearly half of emergency physicians hold a positive attitude toward AI usage, with the most commonly cited advantage being its ability to handle large volumes of clinically relevant, high-quality data in real time. The most frequently cited concern was AI's limitation in delivering reliable decisions in unpredictable clinical scenarios due to insufficient contextual information.

To support successful AI adoption in EM, we recommend a strategic approach that emphasizes reliability, transparency, and sustained physician involvement. Educational programs should focus on improving familiarity and confidence with AI tools among clinicians. Further efforts should also address legal frameworks to clarify accountability, as well as ensure that AI systems remain adaptable and ethically aligned with patient care. Finally, investments in infrastructure and equitable access are crucial to enable AI integration across all healthcare sectors, especially in high-volume public and emergency settings.
